# A CINful way to overcome addiction: how chromosomal instability enables cancer to overcome its oncogene addiction

**DOI:** 10.15252/emmm.202012017

**Published:** 2020-02-18

**Authors:** Daniel Bronder, Samuel F Bakhoum

**Affiliations:** ^1^ Genetics Branch Center for Cancer Research NCI, NIH Bethesda MD USA; ^2^ Division of Cancer Sciences Faculty of Biology, Medicine and Health University of Manchester Manchester UK; ^3^ Human Oncology and Pathogenesis Program Memorial Sloan Kettering Cancer Center New York NY USA; ^4^ Department of Radiation Oncology Memorial Sloan Kettering Cancer Center New York NY USA

**Keywords:** Cancer, Chromatin, Epigenetics, Genomics & Functional Genomics

## Abstract

Oncogene‐addicted tumors present a valuable target for therapeutic intervention and an opportunity to achieve a wide therapeutic window. Nonetheless, resistance to targeted therapies is frequently observed and it arises through multiple mechanisms, including mutations in the target gene. Chromosomal instability, a defining feature of human cancer, has been linked to targeted therapy resistance, but the mechanism underlying this association is poorly understood. In the current issue of *EMBO Molecular Medicine*, Salgueiro *et al* show that chromosomal instability can lead to the generation of alternative oncogenic drivers, thereby providing the ability for cancer cells to overcome the oncogene withdrawal bottleneck. Importantly, this study shows that, by generating *de novo* genomic diversity, chromosomal instability serves as an adaptive response to therapeutic insult.

## Graphical abstract

S. Bakhoum and D. Bronder discuss the study from R. Sotillo *et al* (in this issue of *EMBO Mol Med*) showing that chromosomal instability leads to the generation of alternative oncogenic drivers, and allows tumors to resist the selective pressure of targeted therapy.

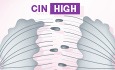

Oncogene addiction is a common phenomenon in human cancers and hence provides an attractive target for cancer therapy. Intervention works on the premise that all cancer cells harbor the mutant or amplified oncogene and are dependent on the oncogenic signaling pathway for proliferation and survival. Drugs like imatinib or gefitinib and erlotinib have heralded a new era of targeted therapies for the *BCR‐ABL* fusion oncogene in Philadelphia chromosome‐rearranged leukemias and non‐small‐cell lung cancers positive for amplified or mutated *EGFR*, respectively (Weinstein & Joe, 2008). Patients respond well initially; unfortunately however, disease recurrence is frequent and directly linked to mutations in structural domains required for drug binding (Gorre *et al*, 2001; Pao *et al*, 2005). This illustrates the severe bottleneck targeted therapies impose upon cancer cells, which these in turn overcome.

Chromosomal instability (CIN) is a process of ongoing missegregation of chromosomes during cell division. CIN is a defining feature of cancer, and it is associated with high intratumor heterogeneity, poor prognosis, metastasis, and drug resistance (Bakhoum & Cantley, 2018; Sansregret *et al*, 2018). It has long been shown that CIN can drive continued tumor growth in response to withdrawal of the initial oncogenic stimulus in mouse models of breast cancer (Sotillo *et al*, 2010). However, the mechanism facilitating continued growth of tumors following oncogene withdrawal has remained unknown.

Here, Salgueiro *et al* (2020) present an elegant study based on their previously described genetically engineered mouse models, seeking to understand the underlying mechanism for the regrowth of tumors following oncogene withdrawal that they previously observed (Rowald *et al*, 2016). Tumors were induced in a doxycycline‐dependent manner in mice that overexpressed a mutated, oncogenic *Kras* allele (K) alone and in combination with the spindle assembly checkpoint gene *Mad2* (KM), which facilitates CIN (Fig 1A). When doxycycline administration was interrupted, most tumors regressed, but 6.6 and 21.3% of K and KM tumors, respectively, did not regress and instead continued growing (Fig 1B). Following this observation, the authors cultured cells from non‐regressed tumors *in vitro* and found that independent of *Mad2* overexpression, the non‐regressed tumor cells displayed high levels of CIN, which exceeded those observed in primary tumors of either genotype (Fig 1C). The non‐regressed tumors also displayed genomic aberrations, which were not detected in primary tumors as evaluated by next‐generation sequencing, again independent of their *Mad2* status.

**Figure 1:**
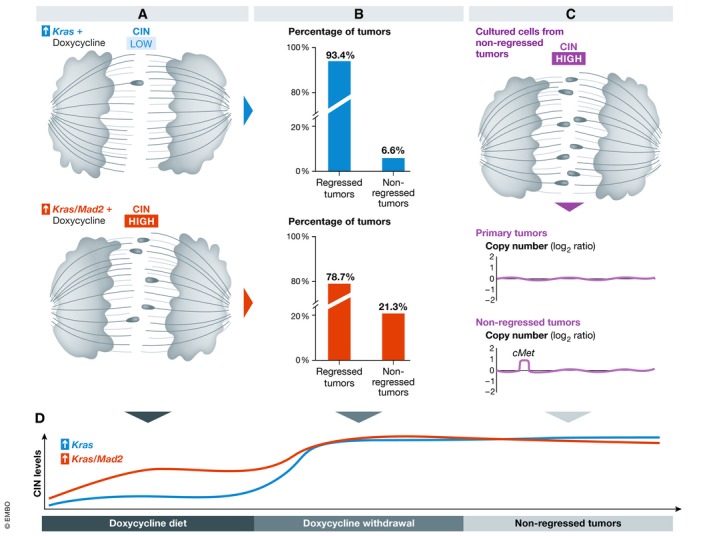
Chromosomal instability promotes oncogene independence in cancer (A) Induction of tumors in *Kras* (K) or *Kras*/*Mad2* (KM) mutant mice resulted in low and high levels of CIN, respectively. (B) Upon doxycycline withdrawal, the majority of tumors regressed, but a minority persisted independent of genotype. Note, the fraction was smaller in K mice. (C) *In vitro* culture of non‐regressed tumors revealed CIN levels even higher than in primary tumors. In addition, the emergence of an amplification along chromosome 6 containing the oncogene *cMet* was noted during genomic analyses in non‐regressed but not primary tumors and also validated functionally. (D) In summary, CIN levels were shown to increase following oncogene withdrawal in the absence of doxycycline beyond what was observed in primary tumors and independent of mice's *Mad2* genotype.

These analyses revealed that a region on mouse chromosome 6, which harbors the oncogene *cMet*, was amplified more frequently in the non‐regressed as compared with the primary tumors (Fig 1C). On a functional level, phosphorylated cMet was only detected in non‐regressed tumors, but not in primary tumors, which further corroborated the genomic data. Using orthogonal approaches, the authors excluded the possibility that a minor fraction of cells in the primary tumor already harbored such an aberration on chromosome 6 involving *cMet*. Thus, they concluded that a gain or an amplification of *cMet* occurs only following *Kras* or *Kras*/*Mad2* withdrawal and continued growth.

Building on the discovery that *cMet* was amplified specifically in non‐regressed tumors, the authors capitalized on the fact that *cMet* can be targeted pharmacologically. Interestingly, primary tumors, which did not harbor cells with amplified *cMet*, were resistant to the *MET* inhibitor tepotinib. In contrast, non‐regressed tumors that harbored the *cMet* amplification were sensitive to tepotinib, and consequently responded to treatment.

This study provides compelling evidence that CIN levels can increase in order to overcome a bottleneck such as oncogene withdrawal (Fig 1D). In patients, genomic alterations that underlie targeted therapy resistance have been observed to occur rapidly after therapy initiation. The observation that CIN can provide tumor cells with an alternative oncogenic pathway is provocative and has important clinical implications. Interestingly, Russo *et al* (2019) observed that colon cancer cells downregulated DNA repair genes and upregulated mutagenic polymerases in response to targeted therapy. Taken together, these data suggest that increasing genomic heterogeneity and mutagenic processes after therapy initiation can provide a substrate for natural selection bypassing the therapeutic bottleneck. This work highlights the importance of longitudinal sampling of tumors prior to and shortly after therapy. While it might be challenging to pinpoint the bypass mechanism for selection in each case in time for clinical intervention, the rapid advances in sequencing technologies and data interpretation might soon enable us to successfully intervene in a subset of cases. Furthermore, it is possible that under given contexts, a relatively limited set of bypass pathways are permissible, therefore enabling a more focused screening approach that would allow rapid intervention.

In summary, this work highlights the importance of CIN in tumor evolution, as well as the salient need to account for genomic copy number alterations—in addition to single‐nucleotide variants—in the clinical practice of oncology. Given the widespread prevalence of CIN in the metastatic setting, this mechanism of therapeutic resistance might be more common than previously thought. It also highlights the need to develop therapeutic approaches to suppress CIN in order to delay the onset of adaptive resistance.

## Conflict of interest

SFB holds a patent related to targeting CIN and the cGAS‐STING pathway in advanced cancer. He owns equity in, receives compensation from, and serves as a consultant and the Scientific Advisory Board and Board of Directors of Volastra Therapeutics Inc. He has also consulted for Sanofi.
